# Supplementary material for: Characterization of Single-Nucleus Electrical Properties by Microfluidic Constriction Channel
Source: Micromachines (Basel). 2019 Oct 31;10(11):740. doi: 10.3390/mi10110740 (PMC6915630; doi:10.3390/mi10110740)
Supplement: Supplementary file 1 [file micromachines-10-00740-s001.pdf]

Table S1. A summary of previously reported electrical parameters of single nuclei.

Nuclear type	Specification	ε_{ne}	C_{ne} (μF/cm²)	R_{ne} (Ω·cm²)	$\sigma_{ne} \times 10^{-3}$ (S/m)	ε_{np}	σ_{np} (S/m)	Reference
Drosophila flavorepleta larvae salivary gland cell			412 ± 62	1.5 ± 0.4				[1]
Drosophila flavorepleta salivary gland cell	In situ		~ 100	3.9 ± 1.4				[2]
	In semi-isolation		~ 100	1.5 ± 0.3				
Drosophila flavorepleta salivary gland cell				1.5 ± 0.4				[3]
Xenopus laevis oocyte				< 0.001				
Triturus viridescens oocyte				< 0.001				
Marine oocyte				< 0.001				[4]
Midge chironomus thummi salivary gland cell	Normal			0.72 ± 0.09				[5]
	Ecdysone			1.38 ± 0.1				
Chironomus salivary gland cell				2				[6]
Murine lymphoma cell (L5178Y)		19 ± 2	0.43 ± 0.03		0.7 ± 0.2	308 ± 20	0.8 ± 0.04	[7]
Mouse liver cell	In situ			0.59 ± 0.33				[8]
	In isolation			0.28 ± 0.23				
Mouse spleen lymphocyte		28	0.62	0.07	6	52	1.35	[9]
Madin-Darby canine kidney cell	Normal			1.4				[10]
	Aldosterone			1.0				
Yeast cell			0.483 ± 0.029		3.22 ± 0.48			[11]
Rat liver cell	Maxwell-wanger theory		1.12 ± 0.20				3.35 ± 1.50	[12]
	Effective medium theory (EMT)		1.16 ± 0.29				2.58 ± 0.54	
	EMT with dipole-dipole interaction		0.92 ± 0.01				1.70 ± 0.10	
Xenopus laevis oocyte				0.13				[13]
Human B-cell	Normal	106 ± 35	2.3 ± 0.69		11.1 ± 7.2		2.04 ± 0.29	[14,15]
	Magala	72.5 ± 11.6	1.6 ± 0.48		3.7 ± 0.9		1.08 ± 0.03	

	Farage	60.3 ± 22.6	1.3 ± 0.39		4.4 ± 2.5		1.07 ± 0.43	
	Raji	79.9 ± 34.4	1.8 ± 0.54		4.0 ± 1.6		1.02 ± 0.25	
	Bjab	108 ± 35	2.4 ± 0.72		2.1 ± 0.7		1.39 ± 0.54	
	Daudi	66.1 ± 7.5	1.5 ± 0.45		2.7 ± 0.3		1.44 ± 0.35	
	Normal	85.6 ± 16.7	1.9 ± 0.57		8.8 ± 0.6		1.26 ± 0.27	
Human T-cell	Peer	61.6 ± 17	1.4 ± 0.42		2.1 ± 0.6		1.42 ± 0.20	
	HDMAR	101.2 ± 55.3	2.2 ± 0.66		3.0 ± 0.2		1.58 ± 0.28	
Eukaryotic cell		6.8 – 100 (41)			0.083 – 7(3)	32 – 300(120)	0.25 – 2.2 (0.95)	[16]
Mammal skeletal myocyte	0.3 GHz microwave<br>1 GHz microwave<br>3 GHz microwave					73.5<br>61.6<br>53.0	3.2<br>3.6<br>6.2	[17]
Human jurkat cell		22.8			4.30	120	0.18	[18]
Human jurkat cell	Normal<br>1 min of pulse<br>20 min of pulse						0.48–0.42<br>0.49–0.31<br>0.31–0.18	[19]
Human jurkat cell	Microfluidic device		1.57 ± 0.01	0.26 ± 0.06			0.63 ± 0.005	[20]
	Time domain reflectometry system		1.19 ± 0.14	0.21 ± 0.02			0.82 ± 0.06	
Human hepatocyte		19.2					1.85	[21]
Human jurkat cell					1.75–2.25			[22]
Human jurkat cell			0.32 ± 0.002	6.37 ± 0.04			37.99 ±8.09	
Mouse B16 cell			0.18 ± 0.11	7.81 ± 4.39			9.16 ± 0.63	[23]
Rat H9C2 cell			0.22 ± 0.05	7.25 ± 0.68			17.2 ± 8.53	

Human PC5 cell			0.16 ± 0.02	5.24 ± 1.98			27.3 ± 4.61	
Human PC6 cell			0.12 ± 0.02	9.26 ± 4.14			8.44 ± 3.48	
Human B16 cell	Normal		1.01 ± 0.56	2.65 ± 2.96			0.35 ± 0.31	
	Gadolinium		1.51 ± 0.51	1.21 ± 0.80			0.57 ± 0.33	
Human H9C2 cell	Normal		1.38 ± 0.13	0.58 ± 0.29			0.44 ± 0.10	
	Gadolinium		1.61 ± 0.08	1.54 ± 0.05			1.20 ± 0.43	
Human PC5 cell	Normal		1.91 ± 0.72	0.37 ± 0.06			0.33 ± .06	
	Gadolinium		1.59±0.08	0.57±0.50			0.26±0.13	
Mouse brain endothelial cell		52			6	52	1.35	[24]
Chinese hamster ovary cell		23.2			1	120	1.50	[25]
Mouse embryonic stem cell	Normal		0.15 ± 0.07		9.9 ± 1.7	1.02 ± 0.01		[26]
	Fixation					1.35 ± 0.05		
	In isolation					1.55 ± 0.45		
	Differentiate at 0 h					0.92 ± 0.20		
	Differentiate at 24 h					1.18 ± 0.40		
	Differentiate at 48 h					1.82 ± 0.16		
